# [Retracted] Correlation of nm 23 expression with pharyngeal cancer and patient prognoses 

**DOI:** 10.3892/ol.2025.15061

**Published:** 2025-04-28

**Authors:** Wenzhong Wei, Xin Wei, Xiao Liang

Oncol Lett 16: 5667–5672, 2018; DOI: 10.3892/ol.2018.9375

Following the publication of this paper, it was drawn to the Editor's attention by a concerned reader that the histological data shown in Fig. 1 on p. 5669, and the nm23 western blot data in Fig. 3 on p. 5670, were strikingly similar to data that had already been accepted for publication in other articles written by different authors at different research institutes. Owing to the fact that the contentious data in the above article had already been published prior to its submission to *Oncology Letters*, the Editor has decided that this paper should be retracted from the Journal. After having been in contact with the authors, they accepted the decision to retract the paper. The Editor apologizes to the readership for any inconvenience caused.

